# Warthin-like and classic papillary thyroid cancer have similar clinical presentation and prognosis

**DOI:** 10.20945/2359-3997000000270

**Published:** 2020-06-19

**Authors:** Roberto Olmos, Francisco Muñoz, Francisca Donoso, Jorge López, María José Bruera, Magdalena Ruiz-Esquide, Lorena Mosso, Nicole Lustig, Antonieta Solar, Nicolás Droppelmann, Pablo H. Montero, Hernán E. González, José Miguel Domínguez

**Affiliations:** 1 Departamento de Endocrinología Facultad de Medicina Pontificia Universidad Católica de Chile Santiago Chile Departamento de Endocrinología, Facultad de Medicina, Pontificia Universidad Católica de Chile, Santiago, Chile; 2 Centro Traslacional de Endocrinología Facultad de Medicina Pontificia Universidad Católica de Chile Santiago Chile Centro Traslacional de Endocrinología (CETREN-UC), Facultad de Medicina, Pontificia Universidad Católica de Chile, Santiago, Chile; 3 Departamento de Anatomía Patológica Facultad de Medicina Pontificia Universidad Católica de Chile Santiago Chile Departamento de Anatomía Patológica, Facultad de Medicina, Pontificia Universidad Católica de Chile, Santiago, Chile; 4 Departamento de Cirugía de Cabeza y Cuello Facultad de Medicina Pontificia Universidad Católica de Chile Santiago Chile Departamento de Cirugía de Cabeza y Cuello, Facultad de Medicina, Pontificia Universidad Católica de Chile, Santiago, Chile; 5 Clínica Las Condes Santiago Chile Clínica Las Condes, Santiago, Chile

**Keywords:** Thyroid cancer, papillary, anti-thyroglobulin antibody, Warthin-like papillary thyroid cancer

## Abstract

**Objective:**

Warthin-like papillary thyroid cancer (WL-PTC) is an uncommon variant of PTC, usually associated with lymphocytic thyroiditis. Scarce evidence suggests that WL-PTC has similar clinical presentation to classic PTC (C-PTC), with no studies comparing risks of recurrence and response to treatment between both variants. Our objective was to describe the clinical presentation and prognosis of WL-PTC and compare it to C-PTC.

**Subjects and methods:**

Retrospective analysis of a prospective cohort, including 370 (96%) patients with C-PTC and 17 (4%) with WL-PTC, consecutively treated with total thyroidectomy with or without RAI, followed for at least 6 months. We compared clinical presentation, risk of mortality and recurrence, as well as response to treatment between both variants.

**Results:**

Of the total cohort: 317 (82%) female, 38 ± 13.5 years, median follow-up 4 years (0.5-28.5); most of them stage I and low/intermediate risk of recurrence. We found no differences regarding clinical-pathological data and risk of recurrence. WL-PTC was associated with a higher rate of anti-thyroglobulin antibodies (TgAb) (65% vs. 36%, p = 0.016) and lymphocytic thyroiditis (59% vs. 34%, p = 0.03). The rates of biochemical and structural incomplete responses were similar in both variants. WL-PTC had a lower rate of excellent response (23% vs. 54%, p = 0.01), which became non-significant when performing analysis by TgAb presence (50% vs. 67%, p = NS).

**Conclusions:**

WL-CPT and C-CPT have similar clinical presentation and rate of recurrence. The lower rate of excellent response to treatment in WL-PTC is due to a higher frequency of TgAb. WL-PCT should not be considered an aggressive variant of PTC.

## INTRODUCTION

Papillary thyroid carcinoma (PTC) is the most common malignant endocrine neoplasia ( 1 ). The classic variant encompasses >90% of PTC, but many other subtypes have been described according to specific histopathological hallmarks. This distinction has clinical implications because some of these variants, including tall cell, solid and columnar, have a more aggressive clinical behavior, consistently associated with less disease-free survival and cancer specific survival ( 2 ).

One of the most recently described PTC subtype is the Warthin-like variant (WL-PTC), first published in 1995 by Apel and cols., and owing its name due to its close resemblance to the papillary lymphomatous cystadenoma, or Warthin tumor of the salivary glands ( 3 ). Histologically, WL-PTC is characterized by oncocytic cells lining the papillae, with a dense lymphoplasmacytic infiltrate of the papillary stalks, usually surrounded by lymphocytic thyroiditis ( 4 , 5 ).

While previously deemed a subtype of the oncocytic variant of PTC, the 4th edition of the World Health Organization (WHO) Classification of Tumors of Endocrine Organs classified WL-PTC as a distinct variant ( 5 ). Although increasingly described by pathologists, WL-PTC is still considered an unusual PTC variant, accounting for 0.2%-1% of PTC, affecting predominantly middle-aged women ( 6 ).

The available published data suggest that WL-PTC has a similar clinical behavior to classic papillary thyroid cancer (C-PTC), with comparable rates of extrathyroidal extension, vascular invasion and nodal metastases ( 6 ). However, to the best of our knowledge, no study has directly compared the risks of recurrence and death, as well as the response to treatment between WL-PTC and C-PTC. Our goal was to analyze the clinical presentation and prognosis between WL-PTC and C-PTC treated in a single institution.

## SUBJECTS AND METHODS

### Subjects

This study was approved by the ethic committee of our institution.

Patients 18 years-old or older, consecutively treated and followed prospectively at our institution between December 2012 and December 2018, who met the following criteria were initially selected: (i) diagnosis of classic or WL-PTC; (ii) subjected to total thyroidectomy, with neck dissection performed only with evidence of clinically apparent nodal disease detected at clinical examination, preoperative neck ultrasound (US), or during intra-operative inspection by the surgeon; and (iii) followed for at least 6 months. Nodal disease was classified in low and high volume according to 2015 American Thyroid Association (ATA) guideline ( 7 ). All biopsies were reviewed by an experienced pathologist (A.S.).

The need for radioiodine (RAI) was defined in each patient following the recommendations of the 2009 ATA guidelines until December 2015, and the 2015 ATA guidelines were followed starting January 2016 ( 7 , 8 ). Patients were classified according to the ATA 2015 recurrence risk category (low, intermediate and high) as well as the eighth edition of the AJCC/UICC staging system (I, II, III and IV) based on the preoperative neck US, intra-operative findings and final surgical pathology report ( 7 , 9 ).

Patients who met any of the following criteria were excluded: (i) partial thyroidectomy; (ii) absence of or incomplete surgical pathology report; (iii) follow-up of less than 6 months; or (iv) Tg, TgAb or neck US not performed during follow-up.

### Follow-up

After initial treatment, all patients were treated with a levothyroxine (LT4) dose sufficient to maintain a serum thyrotropin (TSH) < 1.0 uUI/mL. The patients were followed with clinical examination, Tg, TgAb and neck US every 6 months during the first year and thereafter at 6-12-month interval according to their response to treatment, at the attending physician’s discretion.

### Outcomes

Cervical neck US was performed by experienced radiologists. Negative neck US was defined as either normal exploration or the presence of any of the following findings: (i) thyroid bed avascular nodules ≤ 5mm in diameter, stable during follow-up, or (ii) reactive cervical lymph nodes. Disease recurrence/persistence was defined as suspicious findings at images, confirmed with cytopathologic study and Tg measurement on aspirate washout.

Response to treatment was defined according to US, Tg and TgAb findings during follow-up. Excellent response was defined when all of the following were present: negative US, non-stimulated Tg < 0.2 ng/mL and negative TgAb. Incomplete biochemical response was defined in the presence of negative US and either non-stimulated Tg > 1.0 ng/mL in RAI ablated patients or > 5.0 ng/mL in non-RAI ablated patients, plus negative TgAb, or positive TgAb with an increase ≥ 50% in their concentration. Incomplete structural response was defined as highly suspicious neck US (or any other image modality), confirmed with cytological study, regardless of Tg or TgAb serum concentration. Indeterminate response was defined either as (i) negative neck US plus Tg ≥ 0.2-≤ 1.0 ng/mL in RAI ablated or ≤ 5.0 ng/mL in non RAI ablated patients, plus TgAb(-) or (ii) negative US plus TgAb(+) with decreasing or stable concentration during follow-up (increase < 50% in their concentration) or (iii) non-specific findings on imaging studies ( 10 - 12 ).

### Assays

Tg was measured with a chemiluminescent immunoassay with a functional sensitivity of 0.1 ng/mL (Elecsys II, Roche Diagnostics, Rotkreutz, Switzerland). TgAb was also measured with a chemiluminescent immunoassay (Architect i1000, Abbot Laboratories, Abbott Park, IL), with a reference value of up to 4.11 IU/mL and an analytical sensitivity of 1.0 IU/mL. Both Tg and TgAb were normalized with CRM 457.

### Statistical analysis

Continuous variables are presented either as mean and standard deviation or median with range as appropriate. Categorical comparisons were performed with Fisher’s exact test, and continuous variables were compared using Student’s t-test. A p-value of < 0.05 was considered significant. Statistical analysis was performed using SPSS (v.21.0.0: SPSS, Inc., Chicago, IL).

## RESULTS

Of the 462 patients of this cohort, 387 met inclusion criteria for this study, and 75 were excluded due to lack of follow-up or insufficient clinical and pathological information. Of the included cohort, 317 (82%) patients were female, with a mean age of 38 ± 13.5 years, followed for a median of 4.0 (0.5-28.5) years. Total thyroidectomy alone was performed in 221 (57%) patients, and total thyroidectomy with lymph node dissection in 166 (43%) patients ( Table 1 ).

**Table 1:** Clinical presentation of the 387 included patients

				C-PTC (n = 370)	WL-PTC (n = 17)	P-value
Age (years)				38 ± 13	39 ± 16	NS
Female Gender				301 (81%)	16 (94%)	NS
Length of follow-up (years)				4.1 (0.5-28.5)	2.0 (0.6-6.6)	0.022
Type of surgery	TT TT+LND			212 (57%) 158 (43%)	9 (53%) 8 (47%)	NS
Histology	Diameter (cm)			1.1 (0.1-7.0)	1.5 (0.4-3.0)	NS
	Multifocality		No	230 (62%)	7 (41%)	NS
			Yes	140 (38%)	10 (59%)	NS
	ETE		No	253 (68.3%)	14 (82.4%)	NS
			Microscopic	105 (28.4%)	3 (17.6%)	
			Macroscopic	12 (3.3%)	0 (0%)	
	Lymphovascular invasion			78 (21%)	2 (12%)	NS
	Lymphocytic thyroiditis			125 (34%)	10 (59%)	0.03
Positive TgAb				132 (36%)	11 (65%)	0.016
T	1a			185 (50%)	4 (23.5%)	NS
	1b			120 (32.4%)	8 (47.1%)	
	2			43 (11.6%)	5 (29.4%)	
	3a			5 (1.4%)	0 (0%)	
	3b			4 (1.1%)	0 (0%)	
	4a			13 (3.5%)	0 (0%)	
N	0			206 (55.7%)	10 (58.8%)	NS
	1a			87 (23.5%)	3 (17.6%)	
	1b			77 (20.8%)	4 (23.5%)	
M	0			365 (98.6%)	17 (100%)	NS
	1			5 (1.4%)	0 (0%)	
AJCC VIII	I			357 (96.5%)	16 (94.1%)	NS
	II			10 (2.7%)	1 (5.9%)	
	III			3 (0.8%)	0 (0%)	
ATA risk of recurrence	Low			171 (46.2%)	9 (52.9%)	NS
	Intermediate			178 (48.1%)	8 (47.1%)	
	High			21 (5.7%)	0 (0%)	
RAI administration	Yes			233 (63%)	5 (30%)	0.017
	Dose (mCi)			100 (30-200)	100 (30-150)	NS

C-PTC: classic variant papillary thyroid cancer; WL-PTC: Warthin-like papillary thyroid cancer; TT: total thyroidectomy: TT+LND: total thyroidectomy + lymph node dissection; ETE: extrathyroidal extension; TgAb: Anti thyroglobulin antibodies; RAI: radioactive iodine.

Of the cohort, 370 (96%) had C-PTC, and 17 (4%) had WL-PTC. Fourteen (82%) of 17 WL-PTC were diagnosed since 2015. As shown in table 1 , most patients had stage I PTC, and 95% were classified as low or intermediate risk of recurrence ( Table 1 ). No differences were found in gender, age, extension of surgery, major histopathological features, risk of recurrence and AJCC eighth classification ( Table 1 ).

Compared with C-PTC, WL-PTC was associated with a higher rate of positive TgAb (65% *vs.* 36%, p = 0.016) and lymphocytic thyroiditis (59% *vs.* 34%, p = 0.03). RAI ablation was performed in 66 (35%) of WL-PTC and 238 (64%) of C-PTC (p = 0.017). During the evaluated period, there was a trend towards selective use of RAI: before 2015 it was indicated more commonly (172 of 224, (77%)), in contrast to a more restrictive indication since 2015 (72 of 163 (44%) (p < 0.0001). Since 2015, 68 of 149 (45%) and 4 of 14 (29%) of C-PTC and WL-PTC received RAI, respectively (p NS). The median follow-up was 2.1 (0.6-6.6) years in WL-PTC and 4.1 (0.5-28.5) years in C-PTC patients (p = 0.022) ( Table 1 ).

The rates of both biochemical and structural incomplete responses were similar between WL-PTC and C-PTC ( Table 2 ). During follow-up, 39 (10.5%) patients had incomplete response: 14 (3.8%) biochemical and 25 (6.8%) structural disease. When the analysis was conducted according to the year of diagnosis, in patients treated since 2015, during which RAI administration was similar between both variants, the rate of recurrence remained similar for C-PTC and WL-PTC ( Table 2 ).

**Table 2:** Response to treatment in the whole cohort

		C-PTC (n = 370)	WL-PTC (n = 17)	P-value
Structural or biochemical recurrence	No	331 (89.5%)	17 (100%)	NS
	Yes	39 (10.5%)	0 (0%)	NS
Response to treatment	Excellent	202 (54%)	4 (23%)	0.01
	Indeterminate by Tg o TgAb	100 (27%)	11 (65%)	0.01
	Indeterminate by neck US	29 (8%)	2 (12%)	NS
	Biochemical Incomplete	14 (4%)	0 (0%)	NS
	Structural Incomplete	25 (7%)	0 (0%)	NS

C-PTC: classic variant papillary thyroid cancer; WL-PTC: Warthin-like papillary thyroid cancer; Tg: thyroglobulin; TgAb: anti thyroglobulin antibodies; US: ultrasound.

At the end of follow-up, WL-PTC had a lower rate of excellent response than C-PTC: 4 (23%) *vs.* 202 (54%) (p = 0.01). Interestingly, when subgroup analysis was performed according to the presence of TgAb, this difference became non-significant. In the TgAb negative group, excellent response was achieved in 3 (50%) C-PTC *vs.* 150 (67%) patients with WL-PTC (p = 0.64), whereas in the TgAb positive group, this outcome occurred in 1 (9.1%) C-PTC *vs.* 43 (34%) WL-PTC (p = 0.25) patients ( Table 3 ).

**Table 3:** Response to treatment in the negative anti-thyroglobulin antibodies subgroup

		C-PTC	WL-PTC	P-value
TgAb(-) subgroup	Excellent	150 (67%)	3 (50%)	NS
	Indeterminate by Tg o TgAb	39 (18%)	2 (33%)	NS
	Indeterminate by neck US	14 (6%)	1 (17%)	NS
	Biochemical Incomplete	6 (3%)	0 (0%)	NS
	Structural Incomplete	13 (6%)	0 (0%)	NS

C-PTC: classic variant papillary thyroid cancer; WL-PTC: Warthin-like papillary thyroid cancer; Tg: thyroglobulin; TgAb: anti thyroglobulin antibodies; US: ultrasound.

Of all TgAb(+) patients (n = 143), at the end of follow-up 44 (31%) patients achieved excellent response and in 39 (27%) TgAb concentration decreased ≥ 50% with a negative neck US.

## DISCUSSION

In this study, we compared the clinical presentation and prognosis between WL-PTC and C-PTC and found no differences in the initial risk of recurrence, AJCC staging, as well as biochemical or structural recurrence, after a median follow-up of 4.0 years.

Warthin-like PTC has been a challenging entity since its first description by Apel and cols. in 1995 ( 3 ). Its diagnosis requires an experienced pathologist, because WL-PTC can be mistaken with other oncocyte-rich thyroid neoplasms and lesions, such as the tall cell variant of PTC, Hürthle cell carcinoma and lymphocytic thyroiditis ( 6 ). Moreover, since the publication of the fourth edition of the WHO Classification of Tumors of Endocrine Organs, WL-PTC should also be distinguished from the oncocytic variant of PTC ( 5 ).

With respect to clinical presentation, in our cohort we found no differences between WL-PTC and C-PTC. Like other WL-PTC series, most patients were middle-aged women with early-stage intrathyroidal disease and a low frequency of lymphovascular invasion and lymph node metastases. These findings are consistent with previous reports by Yeo and cols. and Erşen and cols., who described similar characteristics between both variants in diverse ethnicities ( 4 , 6 ).

The most important finding of this study was that WL-PTC and C-PTC had similar rates of both biochemical and structural recurrence. Regarding RAI ablation, it was less frequent in WL-PTC patients, which could be due to the fact that WL-PTC is a recently described variant, and by the fact that many scientific societies currently promote a more individualized management of PTC, endorsing selective use of RAI in an increasing subgroup of patients ( 7 ). For instance, 83% of our WL-PTC patients diagnosed since 2015, a period during which we demonstrated a significantly lower use of RAI. It may be argued that the more frequent use of RAI in C-PTC could have had an impact on its prognosis. However, when considering patients treated since 2015, during which RAI administration was similar between both variants, the rates of recurrence remained non-significantly different.

Regarding response to treatment, WL-PTC had a lower rate of excellent response than C-PTC. According to ATA 2015 guidelines definition, a negative neck US, negative TgAb and a Tg concentration lower than 0.2 ng/dL are required to achieve an excellent response ( 7 ). As previously reported, we found a higher frequency of both of positive TgAb and lymphocytic thyroiditis in WL-PTC compared with C-PTC, which is not surprising considering that since its first description, WL-PTC has been consistently associated with chronic lymphocytic thyroiditis ( 3 , 13 ). Considering the aforementioned conditions, the likelihood of achieving excellent response is reduced by the presence of TgAb. However, when subgroup analysis was performed and only patients with negative TgAb were included, the rates of excellent response were similar in WL-PTC and C-PTC. Moreover, we have previously reported that patients with PTC and positive TgAb, in whom their concentration decrease ≥ 50% and have a negative neck US during follow-up, have a risk of recurrence comparable to patients with excellent response ( 10 ). It is noteworthy that, in our present cohort, nearly a third of TgAb positive patients achieved this specific, excellent-like response, most of which remained free of disease even in absence of RAI ablation.

One strength of this study is that considering that WL-PTC is an infrequent variant of PTC, to the best of our knowledge, this is the first series to report the follow-up and prognosis of patients with WL-PTC and to compare it with C-PTC ( 13 , 14 ). We acknowledge that a median follow-up of 4 years may be deemed short and could result in the underestimation of the risk of disease recurrence. However, 317 of 387 (82%) patients in our cohort achieved either excellent or indeterminate responses due to slightly positive Tg or stable/descending TgAb, both of which have been previously associated with very low risks of death and structural recurrence ( 7 ).

The main limitation of this study is its retrospective nature, similar to most studies in the field of PTC. However, all patients who met the inclusion criteria were followed prospectively and treated according to current ATA and local guidelines. Another limitation may be the shorter length of follow-up in the WL-PTC subgroup, owing to the relatively recent description and inclusion of this variant in thyroid cancer management care holders´ practice.

We conclude that WL-PTC and C-PTC have similar clinical presentation and prognosis, so WL-PTC, although infrequent, should not be considered an aggressive variant of PTC.
